# Anti-Ageism Interventions: An Ecological Approach

**DOI:** 10.1093/geroni/igab046.1668

**Published:** 2021-12-17

**Authors:** Tracey Gendron, Alexa Van Aartjik, Kyrie Carpenter, Ryan Backer, Ashton Applewhite

**Affiliations:** 1 Virginia Commonwealth University, Richmond, Virginia, United States; 2 OldSchool Clearinghouse, San Francisco, California, United States; 3 OldSchool Clearinghouse, Mednham, New Jersey, United States; 4 This Chair Rocks, Brooklyn, New York, United States

## Abstract

Ageism, discrimination based on age, is a systemic problem that occurs at multiple levels of the ecological system – meaning that ageism manifests at the individual, dyadic, institutional and societal levels. The expression of ageism within the levels of the ecological system makes ageism a continually shifting and dynamic force of oppression. Although ageism is a well-documented phenomenon with wide-reaching negative impacts, interventions to mitigate ageism’s effects remain understudied. Little is known about the taxonomy of interventions available addressing ageism at the individual, dyadic, subcultural, institutional and societal levels. The current study conducted a deductive content analysis of an anti-ageism resource clearinghouse, OldSchool.info, to evaluate ageism interventions using an ecological framework. Results indicate the majority of ageism interventions are passive-oriented societal-level macrosystem approaches. A gap analysis will be discussed that indicated more active-oriented interventions with engageable content to address ageism at the personal and relational levels are needed.

